# Comparison of Data Preprocessing Approaches for Applying Deep Learning to Human Activity Recognition in the Context of Industry 4.0

**DOI:** 10.3390/s18072146

**Published:** 2018-07-03

**Authors:** Xiaochen Zheng, Meiqing Wang, Joaquín Ordieres-Meré

**Affiliations:** 1 Department of Industrial Engineering, ETSII, Universidad Politécnica de Madrid, 28006 Madrid, Spain; xiaochen.zheng@alumnos.upm.es; 2 School of Mechanical Engineering and Automation, Beihang University (BUAA), Beijing 100083, China; sy1514206@buaa.edu.cn

**Keywords:** deep learning, data preprocessing, Human Activity Recognition (HAR), Internet of things (IoT), Industry 4.0

## Abstract

According to the Industry 4.0 paradigm, all objects in a factory, including people, are equipped with communication capabilities and integrated into cyber-physical systems (CPS). Human activity recognition (HAR) based on wearable sensors provides a method to connect people to CPS. Deep learning has shown surpassing performance in HAR. Data preprocessing is an important part of deep learning projects and takes up a large part of the whole analytical pipeline. Data segmentation and data transformation are two critical steps of data preprocessing. This study analyzes the impact of segmentation methods on deep learning model performance, and compares four data transformation approaches. An experiment with HAR based on acceleration data from multiple wearable devices was conducted. The multichannel method, which treats the data for the three axes as three overlapped color channels, produced the best performance. The highest overall recognition accuracy achieved was 97.20% for eight daily activities, based on the data from seven wearable sensors, which outperformed most of the other machine learning techniques. Moreover, the multichannel approach was applied to three public datasets and produced satisfying results for multi-source acceleration data. The proposed method can help better analyze workers’ activities and help to integrate people into CPS.

## 1. Introduction

Recent advances in manufacturing industry and Internet of Things (IoT) technology have paved the way for a systematical deployment of cyber-physical systems (CPS), making networked machines perform more efficiently, collaboratively, and resiliently, and transforming manufacturing industries to the Industry 4.0 era [1,2]. According to the Industry 4.0 paradigm, all objects of the factory world are equipped with integrated processing and communication capabilities. This facilitates the vision of the “smart factory”, which enables centralized decision-making while requiring distributed manufacturing equipment and resources [3,4]. More “things”, even people, need to be connected to the system [5]. In contrast to computer-integrated manufacturing (CIM), the Industry 4.0 movement is not gravitating towards workerless production facilities. Instead, people should be integrated into the cyber-physical structure in such a way that their individual skills and talents can be fully realized [6,7].

The development of IoT technology has also promoted the improvement of Human Activity Recognition (HAR), which is based on copious sensors. HAR has been widely applied in surveillance-based security, context-aware computing, ambient assistive living, and assembly tasks analysis [8,9,10,11,12,13,14]. A variety of machine learning algorithms have been used to process human activity data in the big data environment [15,16,17,18,19,20]. In a recently reported study [8], the performance of several common classification methods were compared for recognizing eight daily activities, using the acceleration data collected from wearable sensors in seven different body positions. An overall accuracy of 89% was achieved using the random forest (RF) method, which outperformed artificial neural network (ANN), decision tree (DT), k-nearest neighbors (k-NN), naive Bayes (NB), and support vector machine (SVM) methods.

Deep learning is a paradigm of machine learning that enables computational models consisting of multiple processing layers to learn representations of data with multiple levels of abstraction [21]. Many studies have proven that the use of deep learning can improve the performance of many applications, especially speech and visual object recognition, in addition to many other domains [21,22]. As a powerful feature extraction mechanism, deep learning has also been used to perform HAR in recent years, and significant improvement has been achieved [23,24]. The convolutional neural network (CNN) is one of the most important deep learning approaches that has been used to perform HAR, and has produced satisfying results in a number of studies [25].

Data preprocessing plays an important role in machine learning and deep learning algorithms, and proper preprocessing of data is compulsory for achieving better HAR performance [26,27]. Kotsiantis et al. [26] defined data preprocessing as including data cleaning, normalization, transformation, feature extraction, and selection. Some of the most well-known algorithms for each step of data preprocessing are presented in their study. More specifically, when performing HAR tasks using inertial data from wearable devices, a segmentation operation is necessary, because raw inertial data fluctuate greatly over time. The segmented data should be transformed into proper formats as the inputs of the deep learning models. Spectrograms are a commonly used data preprocessing method for acceleration data. A spectrogram of an inertial signal is a new representation of the signal as a function of frequency and time. Previous studies [23] have shown that spectrogram representation is essential for extracting interpretable features that represent the intensity differences among nearest inertial data points. A method that combines shallow features and those obtained from deep learning models, in order to overcome the defects that resource limitations cause and the simple design of the deep learning models, was proposed in [23]. However, during our experiment it was found that the spectrogram representation of the acceleration signal does not always produce better classification results, and introducing shallow features does not always improve the overall performance, especially when the dataset is sufficiently large and contains multi-source sensor data.

The aim of this study is to compare different data preprocessing approaches for deep leaning supported HAR tasks in different scenarios, like single or multiple sensors, and provide references for future studies. In this paper, a deep learning algorithm was used to classify daily human activities on the basis of the acceleration data that has been provided by wearable devices in different body positions. The study focused on two important steps—data segmentation and data transformation—of preprocessing acceleration data for deep learning algorithms. A comparison among five data segmentation options was undertaken and the impact of segment length on activity recognition accuracy was analyzed. Four different data transformation methods were compared, including raw acceleration data, the multichannel method, the spectrogram method, and the spectrogram integrated with shallow features method. The highest overall recognition accuracy achieved in this study was 99.42% for eight daily activities, based on the data from seven wearable sensors, which outperformed most traditional machine learning techniques. Beside the above-mentioned dataset, the chosen multichannel method was also applied to three public HAR datasets, and the results were compared against existing studies.

## 2. Materials and Methods

**Figure 1:**
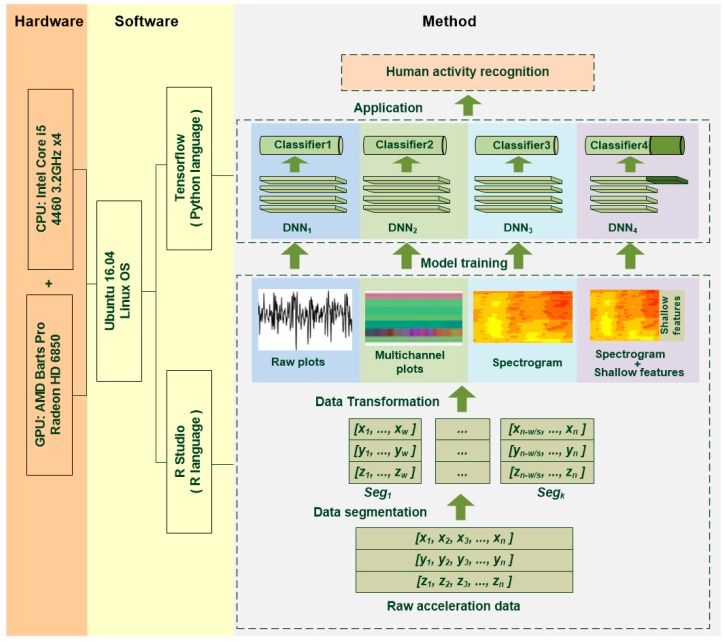
Framework of the proposed method.

The framework of the study is illustrated in Figure 1. The proposed method includes data segmentation, data transformation, deep learning model training, and testing. Human activities are time-dependent, and the raw acceleration data from wearable sensors fluctuates greatly over time, making classification impossible when using a single data point [28]. Most HAR methods are based on a series of data collected in a certain time interval. A segmentation operation is necessary before applying any classification method [8]. The data segments are then transformed into images with four different methods, in order to produce the inputs for the deep learning module. Each input corresponds to a specific deep CNN model and generates a specific classifier. The preprocessed data samples are separated into training and testing samples before the training process. The testing samples are selected randomly. Their quantity depends on the segment options and the total number of samples. More details of each step of the workflow are provided in the following sections.

### 2.1. Data Segmentation

The raw time-dependent acceleration dataset is separated into segments during the data segmentation process. All of the following HAR-related operations, including feature extraction, classification and validation, etc., are based on these segments. The length of the segments depends on the application context and sampling rate of the sensors. Increasing the length of the segments can improve recognition accuracy, but the training time will be increased and more time will be required to obtain sufficient data. This will cause a delay in response for real-time applications [23] and restrict the application scenarios. In most of the existing studies, segments of 1 to 10 s are considered for HAR [29].

### 2.2. Data Transformation

In order to generate the proper inputs for the deep learning models, four different data transformation methods were adopted in this study. These methods transform the raw data segments into different type of representations, from which the deep learning models can extract features automatically. The four methods are explained in detail below.

#### 2.2.1. Raw Plot

**Figure 2:**
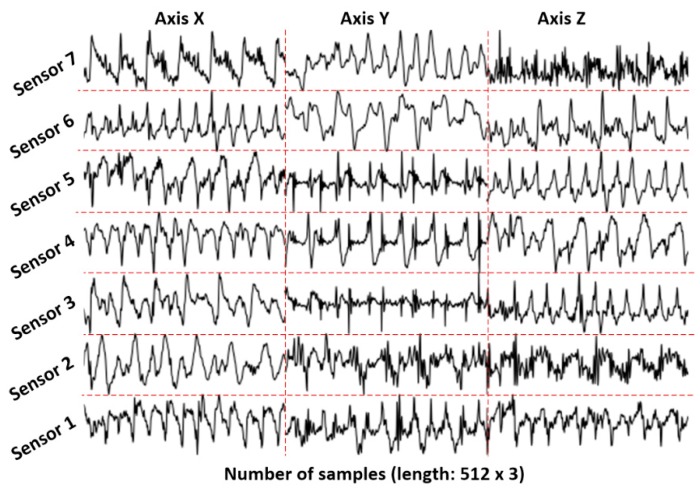
Raw acceleration plot of time domain: segment length 512 and sampling rate 50 Hz (the dotted lines are added manually for better clarification; image resolution is 512 × 512 pixels).

The raw plot method transforms the acceleration data directly to time series images. The three axes are grouped by column, and the data collected from different positions are grouped by row on the same image, if applicable. Both the *x*-axis and *y*-axis resolution of the produced image are the same as the length of the segment, and the color is black and white. For example, Figure 2 shows the image that is generated from an acceleration data segment, which contains 21 separate sub-images that correspond to three axes (by column) and seven sensors (by row). In this plot, the length of the segments represents the number of values included in this segment. The image resolution (512 × 512 pixels) is not related to the lengths (512 × 3) of segments. Higher resolutions may produce better results, but the training time will also increase. This method can represent the temporal acceleration variance. The deep learning models are able to extract activity features based on the intensity and shape of the plot at different locations and on different levels.

#### 2.2.2. Multichannel Method

**Figure 3:**
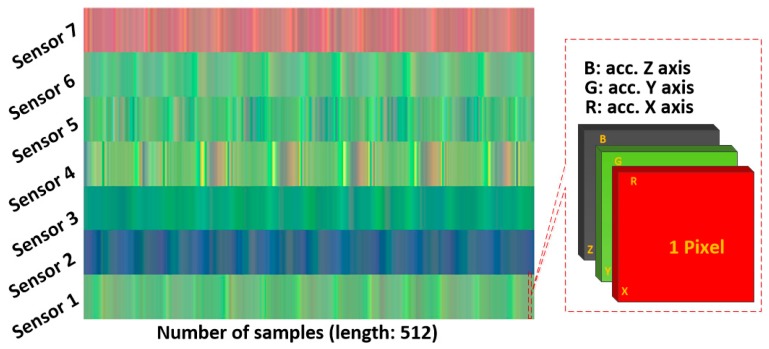
Multichannel RGB color plot on time domain. The segment length is 512 and the sampling rate is 50 Hz (image resolution 512 × 7 pixels).

Unlike the raw plot method, the multichannel method treats the data for the three axes as three overlapped color channels that correspond to red, green, and blue components in the RGB color format. The amplitude of the acceleration signal, which is in the range (−20,20), is projected to a corresponding color value, which is in the range (0,1). In this case, the temporal variance of the acceleration data is transformed into color variance. The three acceleration values of each point are represented as one pixel in the image. The *x*-axis resolution of the image is the same as the length of the segment, and the *y*-axis resolution is the number of sensors. The data collected from different sensors are grouped by row. The advantage of this method is that it reduces the image size enormously and results in a much less training time than the raw plot method. Figure 3 illustrates the principle of this method and an example image produced with this method. The data segment used in this figure is the same as the one used in Figure 2.

#### 2.2.3. Spectrogram

The spectrogram of an inertial signal represents the frequency features of the signal in the time domain. It is the magnitude squared of the short-time Fourier transform (STFT). STFT is used to determine the sinusoidal frequency and phase content of local sections of a signal that changes over time [23,30]. The procedure for computing the spectrogram is to divide a longer time signal into short windows of equal lengths, and then compute the Fourier transform separately for each shorter window. The study by Ravì et al. [23] proved that the spectrogram representation is essential for extracting interpretable features to capture the intensity differences between the nearest inertial data points. The spectrogram representation also provides a form of time and sampling rate invariance. This enables the classification to be more robust against data shifting in time and against changes in the amplitude of the signal and sampling rate.

**Figure 4:**
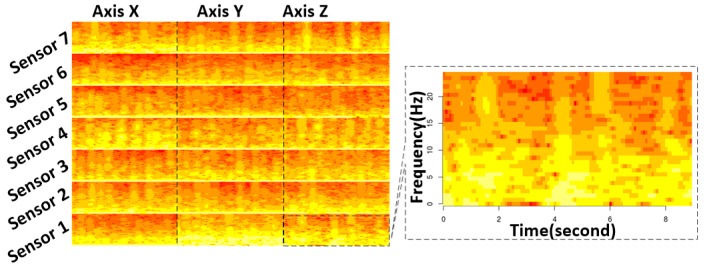
Spectrogram plot of the acceleration data. The segment length is 512 and the sampling rate is 50 Hz, with a short-time Fourier transform (STFT) window length of 64 STFT and an overlap length of 60 STFT (image resolution 336 × 350 pixels).

Figure 4 shows the spectrogram generated from the same data segment that is used in Figure 2 and Figure 3. Here, the resolution of *y*-axis is 350 pixels and the resolution of *x*-axis is determined by the segment length (L), STFT window length (W), and overlap length (P) by the following equation:

Res_x_ = 3 × (L − W)/(W − P), (1)

The spectrograms of different axes and sensors exhibit different patterns. There is also a difference between different activities.

#### 2.2.4. Spectrogram Combined with Shallow Features

Previous studies [23] have shown that when data resources are limited, the features that are derived from a deep learning method are sometimes less discriminating than a complete set of predefined shallow features. To overcome this problem, a method of combining both shallow and deep-learned features was proposed in [23], in order to provide complementary information for the classification. This method was also used in this paper, to compare the results against those of the other three methods. The aim is to determine if this method outperforms other methods for multi-source acceleration data. As suggested in [23], 15 shallow features are extracted from the raw acceleration data of each axis and each sensor, as shown in Table 1. These shallow features are combined with the deep-learned features to form the last layer of the deep CNN model.

### 2.3. Deep Learning Method

**Figure 5:**
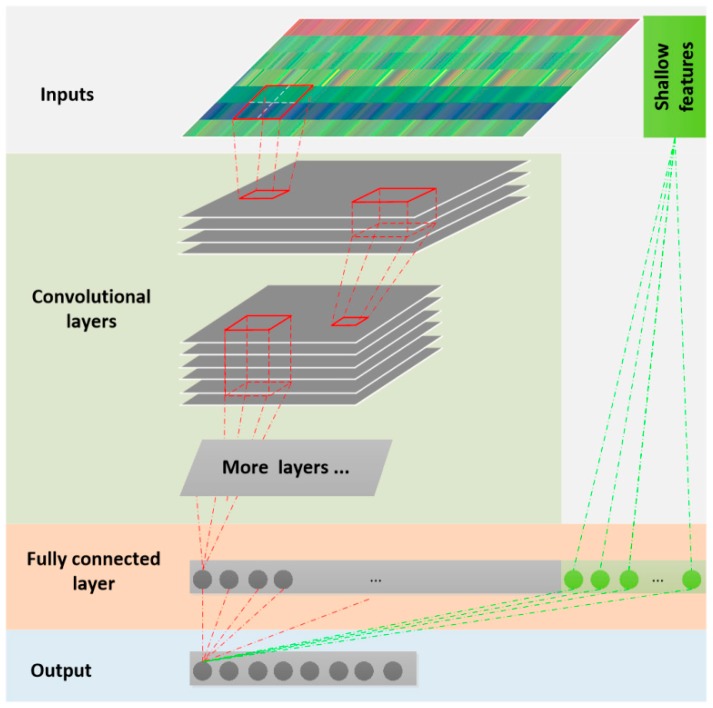
Workflow of deep convolutional neural network (CNN) models.

After preprocessing, the original acceleration data segments are transformed into different types of images, to which the deep learning methods are applied. In this study, the deep CNN algorithm [21,22,23,24,25,26,27,28,29,30,31] is used. Figure 5 shows the overall workflow of the proposed deep CNN method. Different models were built that correspond to the outputs of the four data transformation methods. Each model has its own parameters, such as the number of convolutional layers, the learning rate, pooling size, etc. Following the approach in [23], the shallow features are merged with the deep-learned features on the last fully connected layer, as shown in Figure 5. More details of the deep learning models are available online [32].

## 3. Results

### 3.1. Dataset and Experimental Setup

The dataset contributed by Sztyler et al. [8] was adopted to test the proposed methods. The reasons for this were that it is up-to-date and, according to the authors, is the most complete, realistic, and transparent dataset for on-body position detection that is currently available [8]. This dataset contains the acceleration data of eight activities—climbing stairs down (A1), climbing stairs up (A2), jumping (A3), lying (A4), jogging (A5), standing (A6), sitting (A7), and walking (A8)—of 15 subjects (age 31.9 ± 12.4, height 173.1 ± 6.9, and weight 74.1 ± 13.8, with eight males and seven females). For each activity, the acceleration of seven body positions—chest (P1), forearm (P2), head (P3), shin (P4), thigh (P5), upper arm (P6), and waist (P7)—were recorded simultaneously. The subjects performed each activity for roughly 10 min, except for jumping (about 1.7 min) due to the physical exertion. In total, the dataset covers 1065 min of acceleration data for each position and axes, with a sampling rate of 50 Hz. We filtered and reorganized the dataset to make it suitable for training deep learning models. The detailed processing method and the prepared datasets are available online [32].

As shown in Figure 1, the experiments in this study were implemented with a computer equipped with a four-core Intel Core i5-4460 3.2GHz CPU, an AMD Barts Pro Radeon HD 6850 Graphic Processing Unit (GPU) and a 12 GB of random-access memory (RAM). The operating system is Ubuntu Linux 16.04 64-bit version. Built on top of these is a software combination of RStudio and TensorFlow.

The data preprocessing was performed with RStudio, including data segmentation, data transformation, and shallow feature extracting. The details and complete code is also available in R-markdown format [32]. The deep learning model training and testing were conducted with TensorFlow (Version 1.0), and the model was built in Python (Version 2.7) language. TensorFlow is an interface for expressing machine learning algorithms, and an application for executing such algorithms, including training and inference algorithms for deep neural network models. More specifically, the TF.Learn module of TensorFlow was adopted for creating, configuring, training, and evaluating the deep learning model. TF.Learn is a high-level Python module for distributed machine learning inside TensorFlow. It integrates a wide range of state-of-art machine learning algorithms built on top of TensorFlow’s low-level APIs for small- to large-scale supervised and unsupervised problems. The details of building deep learning models with TensorFlow are provided online, and some of the trained models are also available [32].

### 3.2. Results and Discussion

There are two evaluation schemes for the activity recognition model, which are a person-dependent method and a person-independent, leave-one-out method [17]. For person-dependent evaluation, the data from the same subject are separated to training samples and testing samples. For person-independent evaluation, the data of one or more subjects are excluded from the training process and used for testing. In our study, considering the small number of subjects we have, and in order to compare with a previous study [8], we used the person-dependent method. The classifiers were trained and evaluated for each subject individually. The data of each subject were segmented with a non-overlapping method to avoid over-fitting caused by data duplication in training and testing datasets. Ten percent of the segmented samples were used as testing data, and the remaining samples were used as training data. Sequential selection of samples in time was applied in order to avoid the over-fitting caused by predicting past based on future. All segment lengths were power values of 2 in order to better perform STFT when generating spectrogram images.

These segments were transformed into raw acceleration plots, multichannel plots and spectrogram images, according to the preprocessing methods that were introduced above. For each segment, the 15 shallow features that appear in Table 1 were extracted for each position and axis. Since each segment contains the acceleration data of three axis and seven positions, 315 shallow features were extracted for each segment. The details of data transformation and feature extracting are available [32].

Different deep learning models were built and trained for each combination of the five segmentation options and four data transformation methods. The introduced methods were evaluated for each individual subject. Table 2 presents the aggregated classification results of all 15 subjects, based on different segmentation and transformation combinations. The highest overall accuracy was 97.19%, using the multichannel method based on a segment length of 512 (10.24 s).

The results show that the multichannel method achieved the best performance for all segment lengths. For each of these four transformation methods, the performance improved with the increase of the segment length, from segment length 64 (2.56 s) to 512 (10.24 s). There is an accuracy decrease from segment length 512 (10.24 s) to 1024 (20.48 s). A possible explanation is the significant drop of training sample numbers. The accuracy of the multichannel method is more stable than other methods, among different segment lengths. This means that the performance variance of the multichannel method is less than that of others, and its classification accuracy is less dependent on segment lengths, which implies that this method is more suitable for short-time HAR tasks. Moreover, the introduction of shallow features did not increase performance as expected. In fact, it slightly decreased performance compared to the spectrogram method. One possible explanation is that the number of shallow features, which was 315, was too many, and they were confused with features extracted by the deep learning models.

With the same data preprocessing method, the classification accuracies of different individuals were different due to the variation of data quality, dataset size, and individual behaviors. Table 3 summarizes the overall classification accuracies of the 15 subjects, based on a segment length of 512 (10.24 s) with the four data preprocessing methods. 

Leaving out the impact of the segment length, the four models that were based on the segment length of 512 (10.24 s) were compared in detail. Table 4 presents the classification accuracy of each of the eight activities that the four models produced.

**Figure 6:**
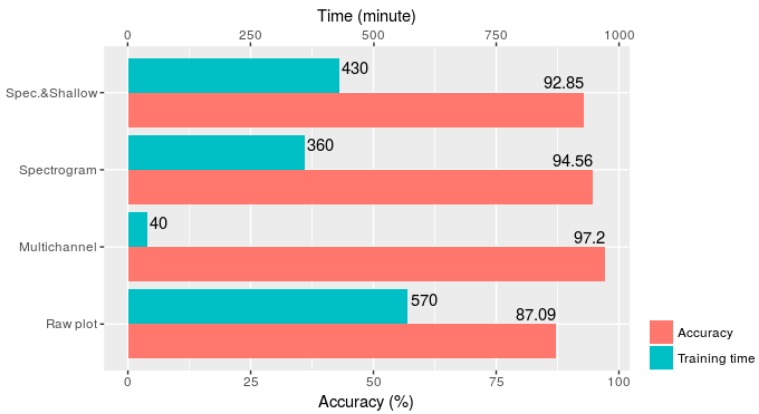
Classification accuracy and training time of the four data transformation methods.

Regarding to training time, the multichannel method also achieved outstanding performance. As shown in Figure 6, the multichannel model took only 40 min to reach the highest accuracy, whereas the other methods required at least 360 min. This proved that the multichannel method provided the best performance, in this case from both accuracy and training time points of view.

Considering the classification accuracy of each activity, the multichannel method perfectly classified the 68 climbing down (A1) samples, as presented in Table 5. It produced a relatively lower accuracy for running activity (A5), where 5 out of 105 running samples were misclassified as standing activity (A7). 

**Figure 7:**
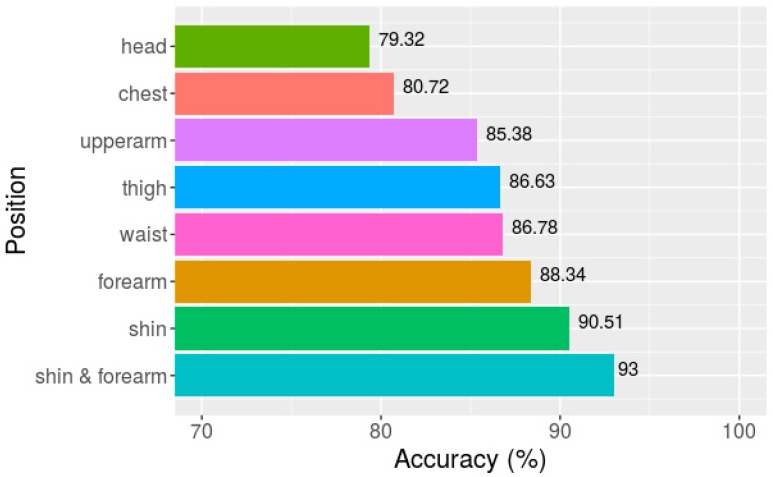
Overall classification accuracies of eight activities based on data from seven single positions and two combined positions.

The classification above is based on the acceleration data that were collected from seven body positions. In real life scenarios, it is difficult to obtain such a complete dataset. Therefore, activity classification using the data from each single position was also undertaken in this study. The combination of segment length 512 (10.24 s) and the multichannel method was used to better compare with the above-mentioned results. Figure 7 shows the overall classification accuracy for the eight activities. The data from the head produced the lowest accuracy (79.32%), whereas the data collected from the shin provided the highest accuracy (90.51%). This result agrees with practical experience that the movements of the head are more stable than other body positions, whereas the movements of the shin are more closely related to different activities, especially to such dynamic ones such as running, jumping, climbing up, and climbing down. By combining the data from the two positions with the data of highest accuracies, the shin and forearm, an overall accuracy of 93.00% was achieved. This is close to the result that was obtained based on the data from all of the seven positions, which was 97.20%.

**Figure 8:**
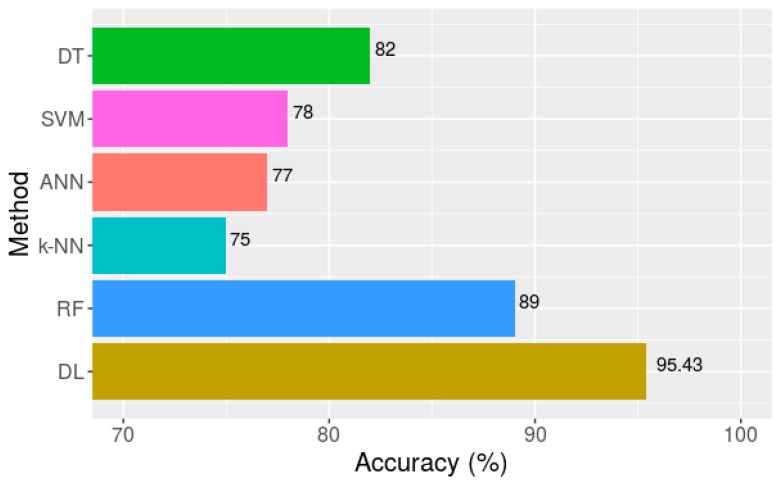
Accuracy of different classification methods [8].

Compared to other traditional classification techniques, such as ANN, DT, k-NN, NB, SVM, and RF, deep learning methods improved the classification accuracy significantly. Figure 8 shows a comparison of the results achieved by the proposed multichannel deep learning method (marked as DL) based on the segment length of 64 (1.28 s) and the results reported in [8], using the same dataset with a similar segment length of one second. It is shown that the deep learning method achieved an overall classification accuracy that was 7.22% higher than RF.

Beside the dataset used above, in order to testify its feasibility, the proposed multichannel data preprocessing method was also applied to another three public HAR datasets, which are WISDM v1.1 (daily activity data collected by a smartphone in a laboratory, with a sampling rate of 20 Hz) [33], WISDM v2.0 (daily activity data collected by a smartphone in an uncontrolled environment, with a sampling rate of 20 Hz) [34,35], and Skoda (manipulative gestures performed in a car maintenance scenario, with sampling rate of 98 Hz) [36]. These datasets were used by Ravì et al. [23], and we used the same segment length as they did, which is a non-overlapping window size of 4 s (for the Skoda dataset) and 10 s (for the WISDM v1.1 and WISDM v2.0 datasets).

The comparison about the per-class precision and recall values obtained by the proposed multichannel transformation method (abbreviated as MCT in the tables) against the results produced by [23] is presented in Table 6. The result shows that the proposed method outperforms the spectrogram integrated with shallow features method in most activities, except the walking and jogging in the WISDM v1.1 dataset and walking in the WISDM v2.0 dataset. In terms of the multi-sensor Skoda dataset, the proposed method perfectly classified most activities, except the open and close left front door activities. This comparison result reveals that the proposed multichannel method is more suitable for multi-source data, although it can also achieve good results for singular sensor data.

## 4. Discussions and Conclusions

In this paper, preprocessing techniques in human activity recognition tasks by deep learning have been considered as a design parameter, and they were shown to be relevant. By comparing different data preprocessing approaches, we came to the following conclusions. Firstly, the length of data segment significantly impacts the final classification accuracy of the deep learning model. The accuracy improves with the increasing of the segment length, and the increasing rate is slower when the segment length is longer. This result agrees with the findings of previous studies that HAR are usually based on data segments of 1 to 10 s. Secondly, four different data transformation methods were compared, and the multichannel method achieved the best performance in both classification accuracy and training time. Unlike the reports of previous studies, we found that the introducing of shallow features did not increase the final accuracy when the experiments were based on a large and multisource dataset. By comparing the classification accuracy based on the data from seven different body positions, it was found that the acceleration data from the shin produced the highest accuracy of 90.51%. A satisfactory accuracy of 93.00% was achieved by combining the data from the shin and forearm. Moreover, we compared the proposed method against some of other common machine learning methods, based on the same dataset, and it was proven that the deep learning method outperforms others impressively. Finally, we applied the proposed multichannel method to three more public datasets, including the car maintenance activity data in a workshop. The results proved that our method can achieve satisfying recognition accuracy. It can help better analyze workers’ activities in a factory environment and help integrate people into the cyber-physical structure in an Industry 4.0 context.

## Figures and Tables

**Table 1:** Shallow features extracted from acceleration data.

Data	Features
Raw signal	max, min, mean, median, variance, kurtosis, skewness, zero-cross, root mean square, standard deviation, interquartile range
First derivative	mean, variance, root mean square, standard deviation

**Table 2:** Overall accuracy (%) of the four data transformation methods, based on five segmentation options.

Segment Length	Raw Plot	Multichannel	Spectrogram	Spectrogram and Shallow Features
64	92.44	94.60	92.86	90.39
128	93.05	96.14	93.37	90.42
256	93.45	96.58	93.94	92.02
512	94.97	97.19	95.56	93.58
1024	82.13	92.81	91.54	85.55

**Table 3:** Variation of overall classification accuracies (%) of 15 subjects based on a segment length of 512 (10.24 s) with four preprocessing methods.

Subject	Raw Plot	Multichannel	Spectrogram	Spectrogram & Shallow Features
Mean	95.25	97.58	95.81	93.92
Min.	92.42	93.91	91.61	88.46
Max.	97.22	99.56	98.57	97.18
Sd.	1.72	2.11	2.35	2.74

**Table 4:** Performance of each model based on a segment length of 512 (10.24 s). A1: climbing down; A2: climbing up; A3: jumping; A4: lying; A5: running; A6: sitting; A7: standing; and A8: walking.

		A1	A2	A3	A4	A5	A6	A7	A8
Raw plot	Precision (%)	97.16	97.99	99.61	99.59	95.18	99.15	92.06	99.49
	Recall (%)	95.41	96.89	98.78	99.18	91.40	98.53	85.24	99.08
	Overall Acc. (%)	94.97			95% CI: (0.9434, 0.9556)				
Multichannel	Precision (%)	97.65	97.96	99.74	99.89	96.29	99.63	96.99	99.72
	Recall (%)	95.56	96.53	99.49	100.00	93.33	99.34	95.04	99.53
	Overall Acc. (%)	97.19			95% CI: (0.9670, 0.9763)				
Spectrogram	Precision (%)	97.65	97.23	99.92	98.60	98.84	97.47	91.18	97.76
	Recall (%)	95.65	96.05	100.00	97.56	98.96	96.55	82.73	96.08
	Overall Acc. (%)	94.56			95% CI: (0.9251, 0.9618)				
Spectrogram & Shallow features	Precision (%)	94.92	98.25	91.51	98.60	95.92	96.60	93.39	95.38
	Recall (%)	91.05	98.59	83.33	97.56	93.14	93.75	88.42	91.51
	Overall Acc. (%)	93.58			95% CI: (0.9157, 0.9512)				

**Table 5:** Confusion matrix generated by the multichannel model based on a segment length of 512 (A1: climbing down; A2: climbing up; A3: jumping; A4: lying; A5: running; A6: sitting; A7: standing; and A8: walking).

Original	Prediction							
	A1	A2	A3	A4	A5	A6	A7	A8
A1	68	0	0	0	0	0	0	0
A2	0	78	2	0	1	0	0	1
A3	0	3	22	0	0	0	0	0
A4	0	0	0	81	1	0	0	0
A5	0	6	0	0	98	1	3	0
A6	0	0	0	0	0	92	1	0
A7	0	0	0	1	5	1	86	0
A8	0	1	0	0	0	0	2	100

**Table 6:** Precision (%) and recall (%) obtained by the proposed multichannel (MCT) method and existing study [23] in three public datasets.

**Dataset 1: WISDM v1.1**							
		**Walking**	**Jogging**	**Sitting**	**Standing**	**Upstairs**	**Downstairs**
Ravì et al. [23]	Prec.	99.37	99.64	97.85	98.15	95.52	94.44
	Rec.	99.37	99.40	98.56	97.25	95.13	95.90
MCT	Prec.	98.34	98.11	100.00	100.00	96.14	98.44
	Rec.	97.31	97.53	100.00	100.00	93.10	97.67
**Dataset 2: WISDM v2.0**							
		**Jogging**	**Lying Down**	**Sitting**	**Stairs**	**Standing**	**Walking**
Ravì et al. [23]	Prec.	98.01	88.65	87.32	85.00	82.05	97.17
	Rec.	97.73	85.85	89.28	76.98	82.11	97.19
MCT	Prec.	98.76	96.85	90.25	87.03	91.02	95.85
	Rec.	97.95	94.96	82.05	75.00	85.94	94.81
**Dataset 3: Skoda**							
		**Write on Notepad**	**Open Hood**	**Close Hood**	**Check Gaps Front**	**Open Left Front Door**	
Ravì et al. [23]	Prec.	96.67	97.78	89.47	91.15	100.00	
	Rec.	91.34	97.78	94.44	92.79	100.00	
MCT	Prec.	100.00	99.54	100.00	100.00	80.00	
	Rec.	100.00	100.00	100.00	100.00	60.00	
		**Close Left Front Door**	**Close Both Left Door**	**Check Trunk Gaps**	**Open and Close Trunk**	**Check Steer Wheel**	
Ravì et al. [23]	Prec.	88.89	92.86	98.78	100.00	93.55	
	Rec.	80.00	94.20	97.59	98.04	100.00	
MCT	Prec.	99.18	100.00	100.00	100.00	94.44	
	Rec.	100.00	100.00	100.00	100.00	88.89	
